# Sulfide-Doped Magnetic Carbon Nanotubes Developed as Adsorbent for Uptake of Tetracycline and Cefixime from Wastewater

**DOI:** 10.3390/nano12203576

**Published:** 2022-10-12

**Authors:** Hassan Sereshti, Elahe Beyrak-Abadi, Mehdi Esmaeili Bidhendi, Irfan Ahmad, Syed Shahabuddin, Hamid Rashidi Nodeh, Nanthini Sridewi, Wan Nazihah Wan Ibrahim

**Affiliations:** 1 Department of Chemistry, Faculty of Science, University of Tehran, Tehran P.O. Box 13145-1384, Iran; 2 School of Environment, College of Engineering, University of Tehran, Tehran P.O. Box 13145-1384, Iran; 3 Department of Clinical Laboratory Sciences, College of Applied Medical Sciences, King Khalid University, Abha 61421, Saudi Arabia; 4 Department of Chemistry, School of Technology, Pandit Deendayal Energy University, Raisan, Gandhinagar, Gujarat 382426, India; 5 Food Technology and Agricultural Products Research Center, Standard Research Institute (SRI), Karaj P.O. Box 31745-139, Iran; 6 Department of Maritime Science and Technology, Faculty of Defence Science and Technology, National Defence University of Malaysia, Kuala Lumpur 57000, Malaysia; 7 Faculty of Applied Sciences, Universiti Teknologi MARA, Shah Alam 40450, Selangor, Malaysia

**Keywords:** antibiotics removal, multi-wall carbon nanotubes, magnetic nanoparticles, cadmium sulfide nanoparticles, adsorption equilibrium

## Abstract

In this study, a magnetic solid-phase extraction method was developed based on multi-wall carbon nanotubes decorated by magnetic nanoparticles (Fe_3_O_4_) and cadmium sulfide nanoparticles (Fe_3_O_4_@MWCNT-CdS) for trace extraction of cefixime and tetracycline antibiotics from urine and drug company wastewater. The adsorbent features were characterized by Fourier transform infrared spectroscopy (FTIR), field emission scanning electron microscope (FESEM), and energy dispersive X-ray analysis (EDX). Various effective parameters on the sorption and desorption cycle, such as sorption time, the mass of adsorbent, pH, salt addition, and material ratio, were investigated and optimized. The data were evaluated using isotherm models, and experimental data were well-fitted to both Langmuir (R^2^ = 0.975) and Freundlich (R^2^ = 0.985) models. Moreover, kinetic of reaction was agreement with pseudo-second-order (R^2^ = 0.999) as compared pseudo-first-order (R^2^ = 0.760). The maximum adsorption capacity for tetracycline and cefixime was achieved at 116.27 and 105.26 mg·g^−1^, respectively. Hence, the prepared adsorbent can be used as an alternative material for enhanced determination of pharmaceutical substances in biological fluids.

## 1. Introduction

Recently, the consumption of antibiotics as medicine has increased significantly, and their usage has grown globally [1]. Cefixime (SF) and tetracycline (TC) are two commonly used antibiotics that can act as potential antibacterial agents against many bacterial infections [2]. Due to their extensive consumption, they produce a large number of pharmaceutical residues that can easily remain in the environment. Additionally, antibiotic residues’ long-term degradation has irreversible effects on human health and the ecosystem and increases bacterial resistance [3]. Thus, it is essential to develop a simple and effective method for the removal of trace amounts of residual antibiotics from wastewater samples.

Recently, traditional strategies and recent technology were combined to develop high-performance methods for efficient removal of antibiotics from water [4], such as adsorption based on carbon materials (granular activated carbon, biochar, carbon nanotube, etc.), zero-valent iron, magnetic separation, filtration, flocculation, column-based MIEX resin ozonation and photocatalytic process, and UV irradiation treatment [5,6,7,8]. Although proposed methods have some advantages, they face some disadvantages, such as being costly, time-consuming, tedious, consuming a large amount of organic solvent, and non-recoverable.

Amongst the aforementioned methods, the techniques based on green and synthetic nanoparticles are the most common approach because the nanoparticles have some significant merits, such as being affordable and having low toxicity, easy operation, high surface area to volume ratio, and high dispersibility [9,10,11,12]. The modification of magnetic nanoparticles improves the dispersibility, enhances surface activity, and prevents agglomeration and oxidation of nanoparticles. Various materials were used to modify magnetic nanoparticles, such as chitosan, alginate, silica, MOF, zeolite, carbon nanotube, graphene, metal oxides, sulfide, etc. [13,14,15,16,17]. Carbon nanotubes (CNTs) have attracted attention in analytical approaches owing to their special features, including a high specific surface area of 3000 m^2^·g^−1^ [18], high susceptibility to form π–π interactions with analytes, chemical stability, thermal conductivity, and easy surface modification [19] as well as CNTs magnetizing easily with iron oxide nanoparticles [20]. Furthermore, MWCNTs provide potential benefits in adsorption approaches and have also been successfully used for antibiotics adsorption [21,22].

Cadmium sulfide (CdS) as a soft nanoparticle (NPs) can increase the possible interactions between adsorbents and antibiotics due to an enhancement in active sites for adsorption [23]. The low-cost CdS NPs possess high surface area and synthesis through simple steps and also provide remarkable potential benefits in water decontamination [24,25,26]. Despite CdS NPs advantages, they tend to aggregate and form larger particle sizes; thus, MWCNTs can prevent this restriction by strongly attaching CdS NPs to the surface of activated MWCNTs [27,28]. This is probably due to the oxygen functionalities on activated MWCNTs (polar surface) that conduct strong interactions with polar CdS NPs. Furthermore, the smooth surface of MWCNTs becomes rougher by rough CdS NPs [29,30].

In this study, MNPs (Fe_3_O_4_) were doped on MWCNTs and functionalized by CdS nanoparticles (Fe_3_O_4_@MWCNTs-CdS) for adsorption of cefixime and tetracycline, the selected antibiotics, in wastewater samples Furthermore, MNPs help to an easy collection of MWCNTs from a large volume of aqueous solution. MWCNTs gain high extraction efficiency through π–π interactions with selected antibiotics and also avoid the CdS NPs aggregation. The presence of CdS improved the adsorption efficacy by introducing various complexation, electrostatic interactions, and H-bonding. Hence, the newly synthesized Fe_3_O_4_@MWCNTs-CdS nanocomposite provided excellent adsorption efficiency for selected antibiotics from wastewater samples.

## 2. Experimental

### 2.1. Reagent and Chemicals

Multi-walled carbon nanotubes (MWCNTs) (purity 80%, outer diameter 10–30 nm and length 100 nm) were purchased from Chengdu Organic Chemicals Co. Ltd. (Chengdu, China), Iron(II) chloride tetrahydrate (FeCl_2_·4H_2_O), Iron(III) chloride hexahydrate (FeCl_3_·H_2_O), sodium hydroxide (NaOH), polyethylene glycol (PEG), cadmium acetate dehydrate (Cd(CH_3_COO)_2_·2H_2_O), methanol (CH_3_OH), sodium thiosulfate pentahydrate (Na_2_S_2_O_3_·5H_2_O), hydrochloride acid (HCl, 37%), nitric acid (HNO_3_, 65%), and sulfuric acid (H_2_SO_4_, 97%) were of analytical grade and supplied by Merck Chemicals (Darmstadt, Germany). The pharmaceutical analytical standards of tetracycline and cefixime were purchased from Solarbio Science & Technology Co., Ltd. (Beijing, China).

### 2.2. Instruments

Morphological and compositional information of the solid product was studied by field-emission scanning electron microscopy and energy-dispersive X-ray spectroscopy (FESEM/EDX) from TESCAN MIRA3 (Brno, Czech Republic). Fourier transform infrared spectrometry (FT-IR) spectra were obtained with KBr pellets in the range 450–4000 cm^−1^ with a Bruker EQUINOX 55 FTIR spectrometer (Bremen, Germany). UV–visible absorption spectra were recorded on RAYLEIGH UV-2601 (Beijing, China) double-beam UV–visible spectrophotometer fitted with a tungsten lamp as the source, volume 3.5 mL quartz cuvette cell (Fisher Scientific, Waltham, MA, USA). Various pH values were measured by WTW Inolab 720 pH meter (Weilheim, Germany).

### 2.3. Synthesis of Fe_3_O_4_@MWCNT

Pristine MWCNTs NPs before magnetization (Fe_3_O_4_) were pre-treated by dispersion in strongly acidic conditions (3:1 molar ratio) that contained HNO_3_ (65%) and H_2_SO_4_ (96%) for 48 h under magnetic stirring at room temperature. Finally, activated MWCNTs were collected and washed several times with acetone and excess distilled water and dried at 85 °C for 24 h [31].

Activated MWCNTs were magnetized as follows: 0.5 g of pre-treated MWCNTs was dispersed in 50 mL distilled water under magnetic stirring. After that, 1.0 g of FeCl_2_·4H_2_O (0.005 mol) and 0.5 g of FeCl_3_·6H_2_O (0.001 mol) were added to the solution and stirred. The solution was heated until 50°C, and an aqueous sodium hydroxide solution (2 M) was added gradually at pH 10. Eventually, the mixture was stirred for 2 h, washed several times with distilled water, and dried at 80°C for 24 h.

### 2.4. Synthesis of Fe_3_O_4_@MWCNTs-CdS Nanocomposite

To further modification of the adsorbent, the cadmium sulfide (CdS) nanoparticles were immobilized on the surface of Fe_3_O_4_@MWCNTs. Then, 5 mmol Cd(CH_3_COO)_2_·2H_2_O, 5 mmol Na_2_S_2_O_3_·5H_2_O, and 10 mL of polyethylene glycol (PEG) were mixed under stirring and heating at 60 °C. Then, 1 g of Fe_3_O_4_@MWCNT was added to the above solution and stirred vigorously for 2 h, and this solution was kept in the oven at 90 °C for 24 h. Thereafter, the obtained Fe_3_O_4_@MWCNTs-CdS was easily separated by an external magnet and washed with excess distilled water, methanol, and ethanol. Then, the separated adsorbent dried in the oven at 85°C for 24 h. In the next step, the CdS nanoparticles (yellow product) were synthesized with the same procedure without the addition of Fe_3_O_4_@MWCNTs into the PEG solution. The characterization and surface morphology of dried CdS nanoparticles and Fe_3_O_4_@MWCNTs-CdS nanocomposite were studied by using FTIR and FESEM/EDS.

### 2.5. Preparation of Samples

Wastewater from the drug company was analyzed as a model of real sample analysis. Wastewater was collected from the vicinity of a pharmaceutical company located in Tehran, Iran. The simulated samples were spiked by the addition of an adequate amount of antibiotics standard solution to give a final concentration of 0.1–100 mg·L^−1^. All samples were stored at 4 °C in the refrigerator.

### 2.6. Magnetic Adsorption Procedure

The experimental adsorption procedure includes subsequent steps: first, 40 mg Fe_3_O_4_@MWCNT-CdS was added to the aqueous samples containing selected antibiotics with different initial concentrations (0.5–10 mg·L^−1^). Then, the mixture was shaken on a plate shaker (350 rpm) for 10–90 min in the different solutions’ pH range of 3–12. Then, the adsorbent was separated from the solution by an external magnetic field that was applied by putting a magnet on the outer tube’s wall. Residual solutions were analyzed by spectrophotometry to measure the residual concentration of antibiotics in the solution and removal percentage.

## 3. Result and Discussion

### 3.1. Characterization

#### 3.1.1. FTIR Spectroscopy

**Figure 1:**
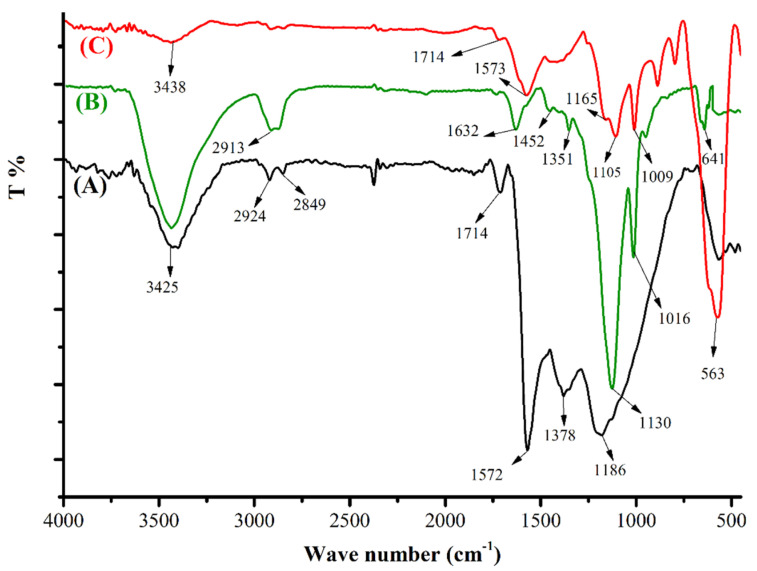
FTIR spectra of (**A**) activated MWNTs, (**B**) CdS nanoparticles, and (**C**) Fe_3_O_4_@MWCNT-CdS nanomaterial.

The FT-IR spectra of the activated MWCNTs, Fe_3_O_4_@MWCNT, and newly fabricated Fe_3_O_4_@MWCNTs-CdS nanocomposite are given in Figure 1. The activated MWCNTs spectrum (Figure 1A) shows a broad band around 3400–3900 cm^−1^ that is attributed to the stretching of O-H. Peaks at 2924 cm^−1^ and 2849 cm^−1^ are ascribed to C-H stretching. The vibrations at 1714 cm^−1^ and 1378 cm^−1^ are assigned to C=O and C-OH, respectively. In Figure 1B, the sharp peak at 563 cm^−1^ refers to Fe-O stretching, which confirmed the magnetization of MWCNT [32]. In Figure 1C, the stretching vibration of the S-H bond appeared at 2900 cm^−1^, and the peaks in the range of 500–600 cm^−1^ correspond to metal–sulfur bonding. Hence, the peaks at 641 cm^−1^ are ascribed to the vibration of the metal sulfide (Cd-S) bands as reported for quantum dots materials [33]. Figure 1C displays the spectra of Fe_3_O_4_@MWCNTs-CdS. In addition to all previous peaks, some additional peaks at 1632 cm^−1^, 1016 cm^−1^, and 641 cm^−1^ are related to the CdS nanoparticles, which confirms the successful coating of CdS nanoparticles onto the surface of Fe_3_O_4_@MWCNT.

#### 3.1.2. FESEM Microscopy

**Figure 2:**
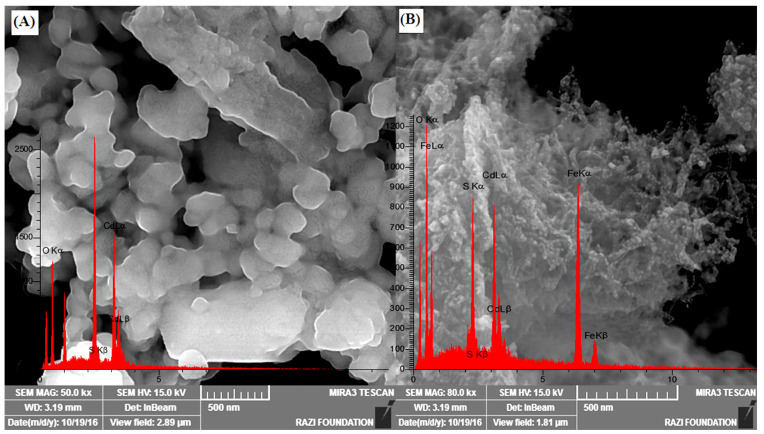
FESEM images and elemental EDX spectra of (**A**) CdS nanoparticles and (**B**) Fe_3_O_4_@MWCNTs-CdS nanocomposite.

Figure 2A depicts the FESEM images of the CdS nanoparticles, and the aggregation of the CdS particles can be observed. The obtained image in Figure 2B depicts that the CdS nanoparticles are well-anchored onto the surface of magnetic MWCNTs. Clearly, the presence of MWCNTs avoids the CdS aggregation; hence, smaller particles were successfully grown on the surface of Fe_3_O_4_@MWCNTs.

#### 3.1.3. EDX Spectroscopy

The elemental composition of the prepared materials was analyzed with the energy-dispersive X-ray spectroscopy (EDX) technique. The EDX spectra for CdS nanoparticles and Fe_3_O_4_@MWCNTs-CdS are illustrated in Figure 2A,B. CdS spectra (EDX) clearly show the elements corresponding to CdS NPs, namely O, Cd, and S. In addition, EDX spectra of Fe_3_O_4_@MWCNTs-CdS nanocomposite confirmed the presence of main elements C, O, Fe, Cd, and S in the backbone of the adsorbent.

### 3.2. Effective Parameters

#### 3.2.1. Material Ratio

**Figure 3:**
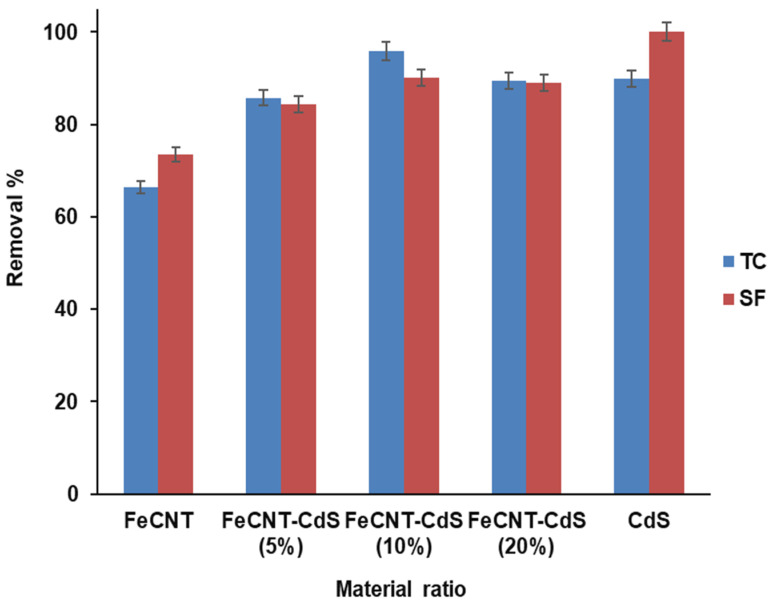
The removal efficiency of different material ratio for TC and SF.

The total removal rate of TC and SF by using Fe CNT, 5% Fe CNT-CdS, 10% Fe CNT- CdS, 20% Fe CNT-CdS, and CdS are demonstrated in Figure 3. This diagram reveals that 20 % Fe CNT-CdS (> 90 removal rate %) has a much higher removal efficiency than other material ratios. The high efficiency attributes to the high surface area, high porosity, and more active sites on the adsorbent surface, resulting in faster transfer of TC and SF to react with the adsorbent and high removal efficiency [34].

#### 3.2.2. Real Sample

A real sample was obtained from pharmaceutical company wastewater. The experiment was conducted for un-spiked and spiked (10 mg·

L^−1^) samples. The cefixime and tetracycline were not detected in the un-spiked wastewater, while detection of TC and SF in the spiked sample was conducted successfully, with a recovery percentage of 91.38 % and 87.44 %, respectively.

#### 3.2.3. pH Effect

**Figure 4:**
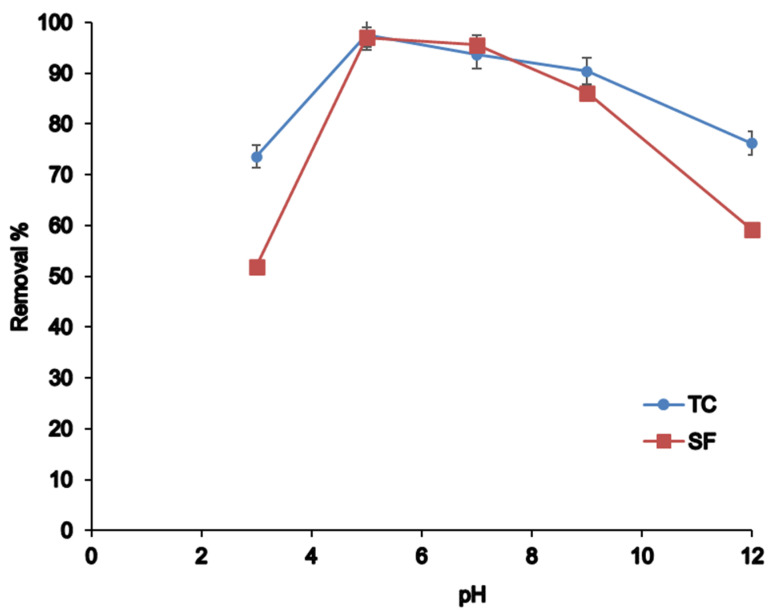
The effect of pH on the adsorption of TC and SF.

pH is an important parameter affecting both analyte and adsorbent surfaces. As illustrated in Figure 4, by increasing pH from 3 to 5, the extraction efficiency increased, then efficiency decreased continually up to pH 11. This can be explained by analyte’s isoelectric points (IEPs) and adsorbent potential zero charge (pH_PZC_). The pH_PZC_ for MWCNTs adsorbent and IEPs for tetracycline were reported in pH 2–4 and pH~6.3, respectively [35,36,37]. Further, tetracycline can be found as different species at different pH, i.e., TCH_3_^+^ at pH < 5, TCH_2_^±^ at pH 5–7, and TCH^−^/TC^2−^ at pH > 7 [38,39]. Thus, high removal efficiency at pH 5–7 can be explained by the positive charge of analytes and the negative charge of adsorbents that increase the electrostatic forces. Additionally, π–π interactions and hydrogen bonding are possible between tetracycline and the adsorbent. Cefixime is a weak acid (pK_a_ 2.5) [40], and due to Le Chatelier’s principle, it can be found in a neutral form in acidic conditions; thus, π–π interactions and hydrogen bonding are the main interactions for adsorption at pH 5. However, the extraction efficiency is decreased at low and high pH, probably due to repulsion forces between protonate/deprotonated analytes and the adsorbent. Moreover, cefixime can hydrolyze at highly acidic and basic pH due to lactam and amide groups [41]. Finally, the highest extraction efficiency for tetracycline and cefixime was obtained at pH 5.

#### 3.2.4. Effect of Adsorbent Amount

**Figure 5:**
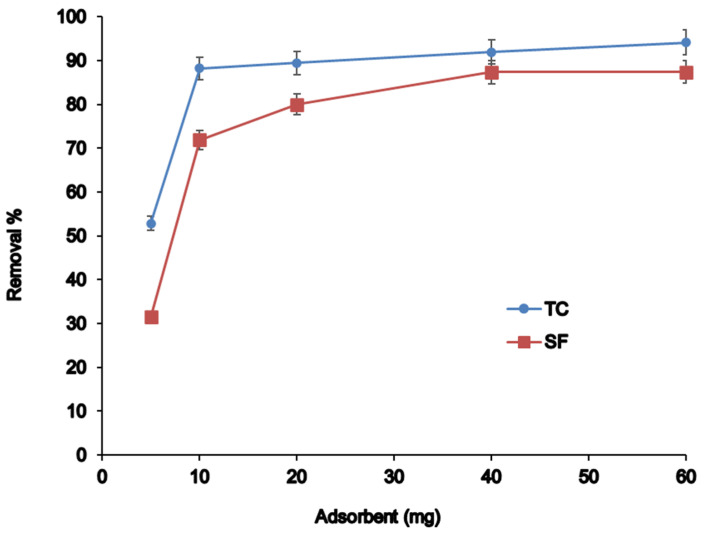
The effect of different amounts of adsorbent dosage.

Different mass of adsorbents in the range of 5–60 mg was carried out for MSPE performance due to the crucial importance of adsorbent amount. The results given in Figure 5 demonstrate that when the amount of adsorbent increased from 5 mg to 40 mg, adsorption capacity enhanced due to excess active sites for predominate interactions. However, after 40 mg, analytes were not available for adsorption, and extraction was almost constant; therefore, 40 mg was selected as the optimum amount of adsorbent [42].

#### 3.2.5. Salt Effect

**Figure 6:**
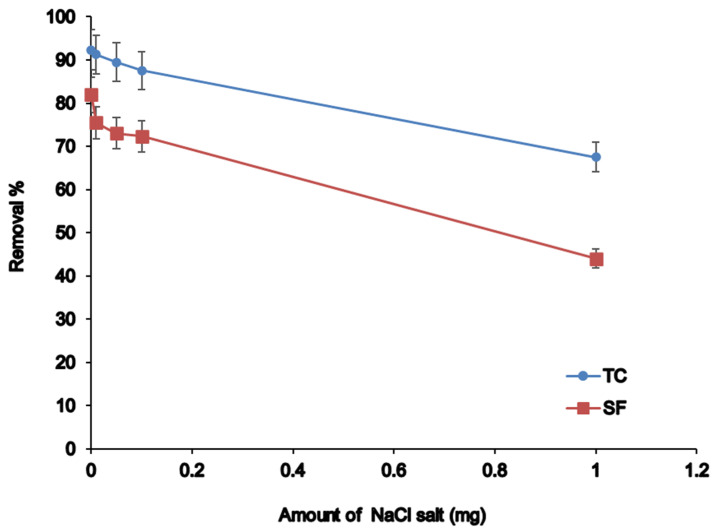
Effect of salt strength on the removal percentage.

Salt augmentation to sample solution can influence extraction efficiency. In this study, the effect of salt concentration was investigated by the addition of different amounts of NaCl salt in the concentration range of 0–10% (*w*/*v*) into the sample solution (Figure 6). As the investigation represented, salt concentration up to 0.5% (*w*/*v*) does not affect the extraction of antibiotics. By increasing salt concentration up to 0.5% (*w*/*v*), the extraction recovery decreased slightly due to the mass transfer of analytes to the surface of the adsorbent or interference with salt ions. In this regard, salt addition is not necessary for our experiments.

#### 3.2.6. Effect of Contact Time

**Figure 7:**
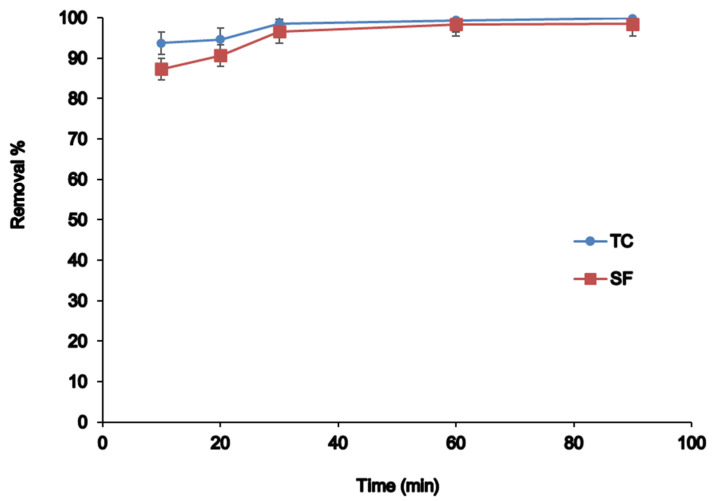
The effect of contact time on removal efficiency.

Contact time can affect the removal efficiency, so different shaking times in the range of 10 to 90 min were investigated. Figure 7 showed the adsorption capacity increased clearly by increasing time in the range of 10 min to 60 min because of sufficient adsorption sites available on the surface of the adsorbent for antibiotics. After 60 min, the absorption remained almost unchanged owing to the occupation of active sites by TC and SF Moreover, after 60 min, there are not probably enough analytes to occupy the active sites; thus, adsorption efficiency is almost constant. Therefore, 60 min was selected as the optimum time for maximum extraction.

### 3.3. Kinetic Study

**Figure 8:**
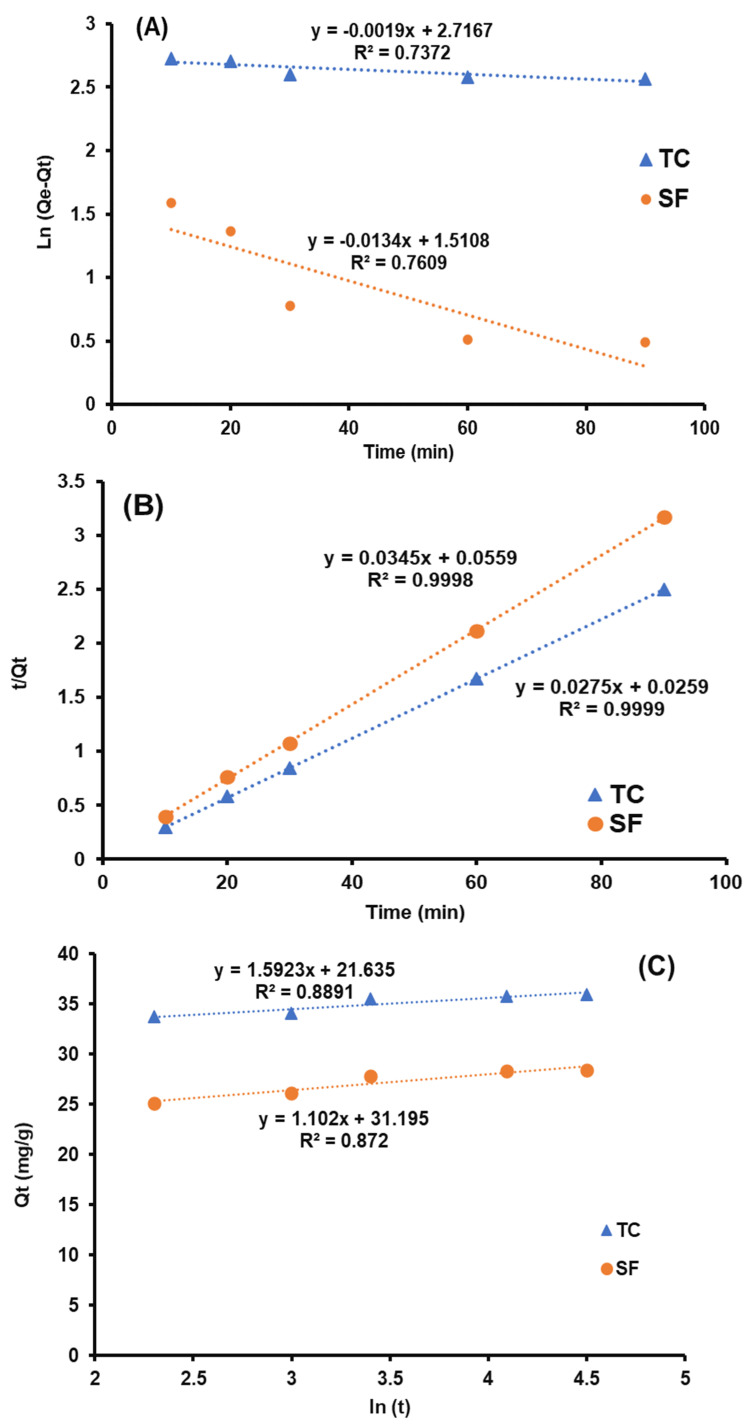
Pseudo-first-order (**A**), pseudo-second-order (**B**), and Elovich (**C**) linear plots.

For the evaluation of adsorption, kinetic pseudo-first-order, pseudo-second-order, and Elovich models, as expressed in Equations (1), (2) and (3), respectively, were studied.

(1) $$\ln(Q_{e}-Q_{t})=\ln Q_{e}-k_{1}t\;\;\;\;$$

(2) $$\frac{t}{Q_{t}}=\frac{1}{k_{2}Q_{e}^{2}}+\frac{t}{Q_{e}}\;\;\;\;\;$$

(3) $$Q_{t}=\left(\frac{1}{\beta }\right)\ln\left(\alpha \beta \right)+\left(\frac{1}{\beta }\right)\ln\left(t\right)$$

where *Q_t_* is adsorption capacity at different times, *Q_e_* is equilibrium adsorption capacity, *k*_1_ is a constant of pseudo-first-order, and *k*_2_ is a pseudo-second-order constant. *α* and *β* (g·mg^−1^) are the Elovich model constant. The values of these parameters can be derived from the slope and intercept that are displayed in Figure 8A–C. According to experimental data as depicted in Table 1, the kinetic of reaction for TC and SF was fitted with a pseudo-second-order model (R^2^ = 0.999 TC, R^2^ = 0.998 SF). In addition, the Elovich model was conducted, and the low values of *R*^2^ (<0.889) demonstrate that the electron-sharing process does not limit the kinetic. In this respect, the adsorption mechanism was conducted through chemisorption.

### 3.4. Isotherm Study

**Figure 9:**
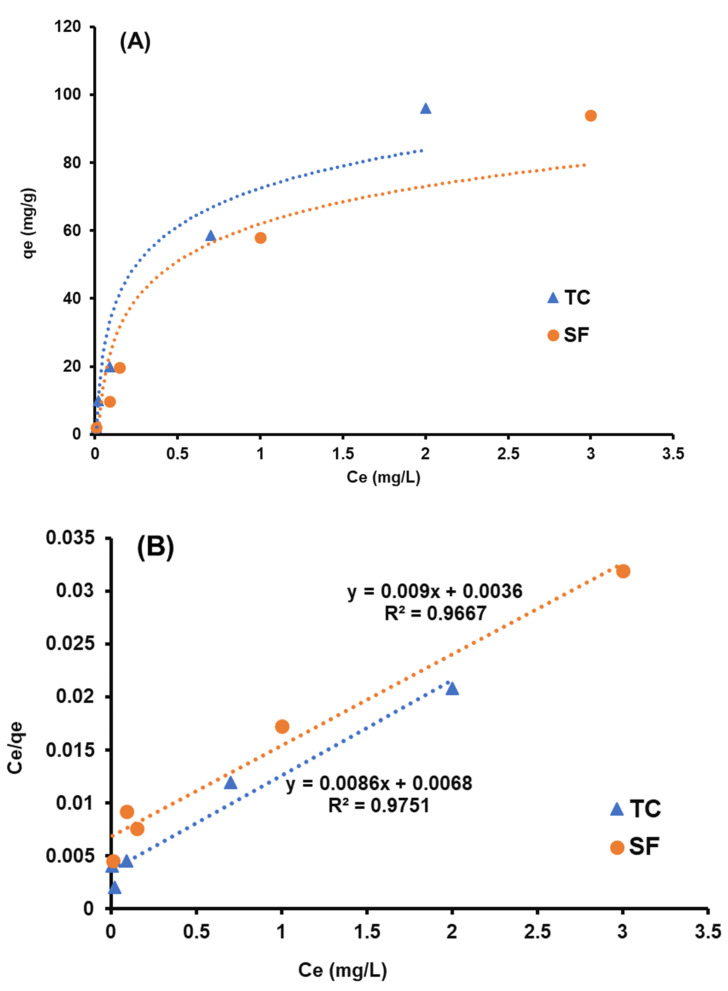

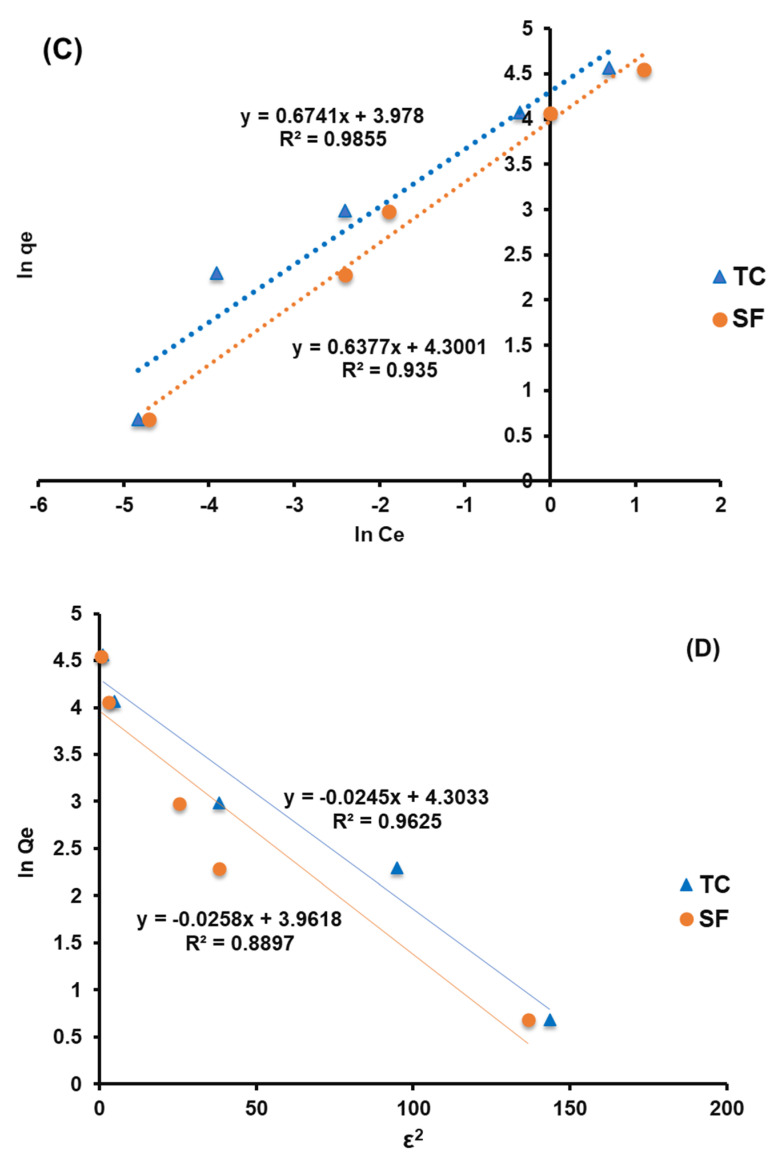
The influence of initial concentration on the adsorption (**A**) equilibrium isotherm, (**B**) Langmuir, (**C**) Freundlich, and (**D**) D-R linear models.

In order to perform the isotherm models, the experimental process is conducted by variation of initial concentrations of TC and SF at constant time. Then, the equilibrium isotherm is plotted in Figure 9A. Hence, Langmuir, Freundlich, and Dubinin–Radushkevich (D-R) isotherm models were utilized to evaluate the isotherm process and prediction of the mechanism of adsorption. The linear form of these models are described in Equations (3), (4) and (5), respectively.

(4) $$\frac{Ce}{Qe}=\frac{Ce}{Qm}+\frac{1}{KQm}$$

(5) $$\ln Qe=\ln Kf+\left(\frac{1}{n}\right)\ln Ce$$

(6) $$\varepsilon =RTLn\left[1+\frac{1}{C_{e}}\right]$$

In these equations, *C_e_* is the equilibrium concentration of TC and SF antibiotics. *Q_e_* (mg·g^−1^) is the experimental equilibrium adsorption capacity, *Q_m_* is the maximum adsorption capacity (mg·g^−1^), *1/n* is the empirical parameter related to the energetic heterogeneity (average energy of sites), and *K_.L._* and *K_.F._* (L·mg^−1^) are the Langmuir and Freundlich equilibrium adsorption constants, respectively. *K_ad_* is constant, and *Qs* is the theoretical adsorption capacity of D-R model (mg·g^−1^), which are related to multilayer adsorption with chemical and physical adsorption onto the heterogeneous surface. The values of the parameters are calculated under linear plots (Figure 9B–D) and listed in Table 2. The values of the parameters (Table 2) perform the adsorption process following the Freundlich and Langmuir models due to high *R*^2^ > 0.91 for both models. Langmuir’s model justifies the formation of a monolayer with physical and chemical mechanisms on the homogenous surface of the adsorbent. In contrast, Freundlich’s model expresses the formation of multilayer adsorption and physical adsorption. The evaluation of experimental data shows that the mechanism of adsorption of TC and SF on the surface of Fe-CNT-CdS is in agreement with both Langmuir and Freundlich models. Hence, the D-R model was conducted, and the high value of *R*^2^ (0.962 and 0.889) confirms that the adsorption pattern is following a multilayer process.

### 3.5. Effect of Temperature and Thermodynamic

Thermodynamic parameters such as ΔG (free energy change), *K_.D._ = Q_e/_C_e_* (thermodynamic constant), ΔH (enthalpy), and ΔS (entropy) can be expressed by Equations (6) and (7). The obtained calculations from the experimental (Table 3) determined the spontaneous physical and chemical mechanism in parallel with increasing temperature (ΔG < −40 KJ/mol). Additionally, the positive enthalpy revealed the endothermic adsorption process of TC and SF.

(7) $$\Delta G{}^{\circ}=-RT\ln K_{D}\;$$

(8) $$\ln K_{D}=-\frac{\Delta H{}^{\circ}}{RT}+\ln\frac{\Delta S{}^{\circ}}{R}\;$$

### 3.6. Reusability and Stability

The stability of the adsorbent was evaluated under a continuous adsorption-desorption reusability process. After each adsorption process, the adsorbent was separated with an external magnet and washed with 6 mL methanol three times. Then, adsorbent was dried, and we proceeded to investigate their reusability for five cycles. The adsorbent was provided appropriate stability since the removal efficiency (first cycle > 0.91 %) was not changed significantly after five adsorption-desorption cycles (first cycle > 0.84 %).

### 3.7. Adsorption Mechanism

The two selected antibiotic molecules (TC and SF) possess three functional groups, including hydroxide (-OH), amine (-NH), and carbonyl (COO-). Furthermore, Fe-CNT-CdS adsorbent possesses various active sites on the surface, including -OH and COO-, sulfur, cadmium, and iron oxide. The proposed functional groups of TC and SF and active adsorbent sites (S2-, M+, π-stacking of CNTs, and O-) can attract each other. Thus, the attraction is involved with the electrostatic interaction. Hydrogen binding and π–π interaction occur between the antibiotic’s molecules and magnetic adsorbent.

### 3.8. Comparison

Table 4 reports the adsorption capacity of several adsorbents for removing tetracycline or cefixime at different pH. According to data, the function of synthetic Fe-CNT-CdS is superior to other adsorbents. The high performance of the current adsorbent is associated with high porosity and functional groups on the surface, which enhance the removal percentage of tetracycline and cefixime from environmental aqueous.

## 4. Conclusions

In this study, CdS NPs were decorated on magnetic multi-walled carbon nanotubes as novel nanocomposite (Fe_3_O_4_@MWCNTs-CdS) via a simple procedure. This nanocomposite was used as an adsorbent to remove cefixime and tetracycline antibiotics from drug company wastewater and biological urine fluid. Isotherm models, including Langmuir and Freundlich models, were investigated. The experimental data were in agreement with the Langmuir model, which suggests a monolayer of tetracycline and cefixime onto the adsorbent and provides adsorption capacity 116.27 mg·g^−1^ and 105.26 mg·g^−1^, which indicates high potential adsorbent in comparison with other published works in the treatment of TC and SF The kinetic mechanism was examined using pseudo-first-order and pseudo-second-order models. The experimental data in the current study were fitted with a pseudo-second-order model (R^2^ = 0.999). The proposed method is environmentally friendly due to the lack of toxic organic solvents.

## Figures and Tables

**Table 1:** Kinetic model constants for TC and SF adsorption using Fe-CNT-CdS.

Kinetic Models	Model Constants	T.C.	S.F.
	*k_1_* (min^−1^)	0.001	0.013
Pseudo-first order	*q_e_* (mg·g^−1^)	14.61	4.43
	*R* ^2^	0.732	0.699
	*k_2_* (g mg^−1^ min^−1^)	0.007	0.001
Pseudo-second order	*q_e_* (mg·g^−1^)	37.03	29.41
	*R* ^2^	0.999	0.999
	*α*	13.61	28.65
Elovich	*β*	0.61	0.90
	*R* ^2^	0.889	0.872

**Table 2:** Isotherm models for TC and SF adsorption from aqueous media.

Models	Parameters	T.C.	S.F.
Langmuir	*Q_m_* (mg·g^–1^)	116.27	105.26
	*R* ^2^	0.971	0.966
	*K_L_* (min^–1^)	1.26	3.06
Freundlich	*K_F_* [(mg·g^−1^) (L/mg)]^1/*n*^	48.59	71.59
	*n*	1.49	0.93
	*R* ^2^	0.935	0.985
	*K_ad_*	0.025	0.024
D-R	*R* ^2^	0.962	0.900
	*Q_s_* (mg·g^–1^)	72.31	51.07

**Table 3:** Different thermodynamic parameters.

Adsorbate	Temp. °C	qe (mg/g)	∆G (KJ/mol)	∆H (KJ/mol.K)	∆S (KJ/mol.K)
	25	59.92	−35.23		
TC	30	59.88	−34.79	−40.69	−0.018
	40	59.82	−34.88		
	25	58.01	−27.17		
SF	30	57.68	−27.24	−21.77	−0.017
	40	57.03	−27.44		

**Table 4:** Comparison of adsorption capacity of different adsorbents.

Adsorbent	Adsorbate	Q_m_ (mg·g)	pH	Reference
Chitosan 10B	Cefixime	37.04	5	[43]
MGO-chitosan	Cefixime	30.80	8	[44]
CNT/cyclodexine/MnFeO	Tetracycline	40.36	7	[45]
biochar	Tetracycline	58.47	5.1	[46]
Polyelectrolyte-modified nanosilica	Cefixime	10.40	4	[42]
Fe-CNT-CdS	Tetracycline-cefixime	116.27 105.26	5	This study

## Data Availability

Not applicable.
